# Hypoxic hUCMSC-derived extracellular vesicles attenuate allergic airway inflammation and airway remodeling in chronic asthma mice

**DOI:** 10.1186/s13287-020-02072-0

**Published:** 2021-01-06

**Authors:** Liyang Dong, Ying Wang, Tingting Zheng, Yanan Pu, Yongbin Ma, Xin Qi, Wenzhe Zhang, Fei Xue, Zirui Shan, Jiameng Liu, Xuefeng Wang, Chaoming Mao

**Affiliations:** 1 Department of Nuclear Medicine, The Affiliated Hospital of Jiangsu University, Zhenjiang, Jiangsu 212000 People’s Republic of China; 2 Department of Respiratory Diseases, The Affiliated Huai’an Hospital of Xuzhou Medical University, Huai’an, Jiangsu 223002 People’s Republic of China; 3 Jiangsu Key Laboratory of Pathogen Biology, Department of Pathogen Biology and Immunology, Nanjing Medical University, Nanjing, Jiangsu 211166 People’s Republic of China; 4 Department of Neurology Laboratory, The Affiliated Jintan Hospital of Jiangsu University, Jintan, Jiangsu 213200 People’s Republic of China; 5 Department of Central Laboratory, The Affiliated Hospital of Jiangsu University, Zhenjiang, Jiangsu 212000 People’s Republic of China

**Keywords:** Hypoxia, Human umbilical cord mesenchymal stem cells, Extracellular vesicles, Lung injury, Asthma

## Abstract

**Background:**

As one of the main functional forms of mesenchymal stem cells (MSCs), MSC-derived extracellular vesicles (MSC-EVs) have shown an alternative therapeutic option in experimental models of allergic asthma. Oxygen concentration plays an important role in the self-renewal, proliferation, and EV release of MSCs and a recent study found that the anti-asthma effect of MSCs was enhanced by culture in hypoxic conditions. However, the potential of hypoxic MSC-derived EVs (Hypo-EVs) in asthma is still unknown.

**Methods:**

BALB/c female mice were sensitized and challenged with ovalbumin (OVA), and each group received PBS, normoxic human umbilical cord MSC-EVs (Nor-EVs), or Hypo-EVs weekly. After treatment, the animals were euthanized, and their lungs and bronchoalveolar lavage fluid (BALF) were collected. With the use of hematoxylin and eosin (HE), periodic acid-Schiff (PAS) and Masson’s trichrome staining, enzyme-linked immune sorbent assay (ELISA), Western blot analysis, and real-time PCR, the inflammation and collagen fiber content of airways and lung parenchyma were investigated.

**Results:**

Hypoxic environment can promote human umbilical cord MSCs (hUCMSCs) to release more EVs. In OVA animals, the administration of Nor-EVs or Hypo-EVs significantly ameliorated the BALF total cells, eosinophils, and pro-inflammatory mediators (IL-4 and IL-13) in asthmatic mice. Moreover, Hypo-EVs were generally more potent than Nor-EVs in suppressing airway inflammation in asthmatic mice. Compared with Nor-EVs, Hypo-EVs further prevented mouse chronic allergic airway remodeling, concomitant with the decreased expression of pro-fibrogenic markers α-smooth muscle actin (α-SMA), collagen-1, and TGF-β1-p-smad2/3 signaling pathway. In vitro, Hypo-EVs decreased the expression of p-smad2/3, α-SMA, and collagen-1 in HLF-1 cells (human lung fibroblasts) stimulated by TGF-β1. In addition, we showed that miR-146a-5p was enriched in Hypo-EVs compared with that in Nor-EVs, and Hypo-EV administration unregulated the miR-146a-5p expression both in asthma mice lung tissues and in TGF-β1-treated HLF-1. More importantly, decreased miR-146a-5p expression in Hypo-EVs impaired Hypo-EV-mediated lung protection in OVA mice.

**Conclusion:**

Our findings provided the first evidence that hypoxic hUCMSC-derived EVs attenuated allergic airway inflammation and airway remodeling in chronic asthma mice, potentially creating new avenues for the treatment of asthma.

## Background

Asthma is a chronic respiratory disease that affects more than 300 million people worldwide, and its prevalence continues to increase every year [1]. Airway inflammation and remodeling is the fundamental element of asthma, and they induce histological changes in the airway structure, including the thickening of airway basement membranes, smooth muscle proliferation, and increased fibrosis, which results in lung function decline [2]. Although inhaled corticosteroids are widely used to control asthma, these approaches do not reverse the ongoing remodeling process [3]. Novel therapeutic strategies are still required.

In recent years, studies have shown that mesenchymal stem cells (MSCs) exhibit obviously inhibitory effects not only on airway inflammation but also on airway remodeling in various experimental asthma models [4]. The secreted biological factors are a key mechanism of action of MSCs [5]. Extracellular vesicles (EVs) are small membrane vesicles (50 to 200 nm) released by almost all cell types, which contribute to donor cell-mediated biological effects by transferring a subset of proteins, lipids, and nucleic acids [6]. Transplantation of MSC-EVs or MSCs exhibits similar therapeutic effects through reduction of collagen fiber content and inflammation in lung tissue in chronic experimental allergic asthma [7], suggesting that MSC-EVs are a major kind of functional forms of MSCs [8]. In addition, MSC-EVs are less complex, small, better defined, and easy to store and have stable biological function; thus, they have less potential side effects than MSC therapies and can even be considered an off-the-shelf product [9]. Obviously, MSC-EVs represent a promising therapeutic strategy for asthma disease [7, 10–13].

During in vitro culture, MSCs are generally exposed to normoxic conditions (21% O_2_). In fact, most MSCs exist in an environment of 2–8% oxygen in the body [14]. Hypoxic preconditioning not only improves proliferation, differentiation, migration, and EV secretion of MSCs but also exhibits therapeutic potential in many kinds of disease models [15–17]. Recently, Kwak et al. [18] found that in a mouse model of asthma induced by ovalbumin (OVA), cobalt chloride (hypoxia-mimetic compound) enhances the anti-inflammatory potency of human umbilical cord blood-MSC through hypoxia inducible factors 1α (HIF-1α)-miR-146a-mediated signaling pathway. Based on hypoxic conditions reflecting the living environment of MSCs and strengthening their therapeutic effects, we hypothesized that the anti-asthma effect of MSC-EVs might be enhanced by culture in hypoxic conditions.

In this study, we extracted EVs from nomoxic or hypoxic hUCMSCs (human umbilical cord MSCs; normoxic hUCMSC-EVs (Nor-EVs) or hypoxic hUCMSC-derived EVs (Hypo-EVs)) and then comparatively investigated their therapeutic potential in a mouse model of OVA-induced allergic inflammation and airway remodeling. We also sought to determine the possible associated mechanisms of Hypo-EVs’ effectiveness.

## Materials and methods

### Cell culture

Human umbilical cord samples were obtained from informed, consenting mothers at the Affiliated Hospital of Jiangsu University (Zhenjiang, China). Human umbilical cord MSCs (hUCMSCs) were isolated from fresh umbilical cord samples as previously described [19] and maintained in stem cell culture medium (Cyagen, Guangzhou, China) at 37 °C with 5% CO_2_. HLF-1 (Human lung fibroblast cells) were purchased from the Cell Bank of Chinese Academy of Sciences and cultured with DMEM medium (Gibco, Carlsbad, CA) containing 10% FBS (Gibco) and 1% pen/strep (Gibco) at 37 °C with 5% CO_2_.

### Generation and characterization of extracellular vesicles (EVs)

HUCMSCs were seed at a density of 2 × 10^6^ cells/100 mm dish and cultured for 24 h under hypoxic (5% O_2_) or normoxic (21% O_2_) conditions in serum-free culture medium. The hUCMSC-EVs were isolated from the supernatant of hUCMSCs by ultracentrifugation (Beckman Coulter Optima L-100 XP ultracentrifuge, Miami, FL) as previously described [19]. Briefly, the hUCMSC culture supernatants were centrifuged at 300×*g* for 10 min and 2000×*g* for 20 min to remove cell debris. Then, the supernatants were collected and subjected to ultracentrifugation at 100,000×*g* for 90 min at 4 °C. After that, the pellets were gathered, washed, and resuspensed in PBS.

Morphology of the EVs was observed using transmission electron microscopy (JEM-1200EX; JEOL Ltd., Tokyo, Japan). The protein content of the concentrated EVs was detected using BCA protein assay kit (Beyotime, Nantong, China). The particle size distribution of the EVs were determined by nanoparticle trafficking analysis using NanoSight NS300 (Malvern Instruments Ltd., Worcestershire, UK) according to the manufacturer’s manual.

### Cell proliferation and viability assay

hUCMSCs (5 × 10^3^) in 96-well plates were exposed to hypoxia for 24 h (normoxic cells served as control). Cell proliferation and viability were determined using a BrdU Cell Proliferation Assay Kit (BioVision, Milpitas, CA) and cell counting kit-8 (KeyGEN BioTECH, Nanjing, China) respectively, according to the manufacturer’s manual.

### Cell transfection

The hUCMSCs were transfected with synthetic miR-146a-5p inhibitor or negative control (100 nM; GenePharma, Shanghai, China) using Lipofectamine 2000 (Invitrogen, Carlsbad, CA) according to the manufacturer’s procedures. Then, the EVs were isolated using the protocol described before, and called miR146a^KD^-Hypo-EVs and NC-Hypo-EVs, respectively.

### Mouse model of chronic allergic airway inflammation

Six-week-old female BALB/c mice were purchased from the Comparative Medicine Centre of Yangzhou University (Yangzhou, China). The mice were randomly assigned to 1 of the 4 following groups (*n* = 6 mice per group): control, PBS, Nor-EVs, and Hypo-EVs. Chronic experimental asthma was induced according to previous reports [20, 21]. Briefly, apart from the control group, the mice were sensitized with 40 μg OVA ovalbumin (OVA, Sigma, Poole, UK) and 2 mg 10% aluminum hydroxide (Sigma) in PBS on day 0, 7, and 14 by intraperitoneal injection. From days 21 to 55, the sensitized mice were challenged with aerosolized OVA (5%) for 20 min in PBS as previously described [22]. Bronchoprovocation was performed in a vented plastic chamber (30 × 20 × 15 cm). Aerosol particles with a diameter of approximately 3–5 μm were created from an ultrasonic nebulizer (403 M; YUWELL, Zhenjiang, China), directed into the plastic chamber, and vented to a fume hood. After aerosolizing, the mice were intranasally infused with 20 μL OVA (40 mg/mL) (three times a week). A therapeutic regimen was instigated by injecting 0.1 mL PBS (PBS group), Nor-EV, or Hypo-EV (40 μg [7] suspended in 0.1 mL PBS; intravenous; Nor-EVs group or Hypo-EVs group) on day 26, and after four times treatment (day 26, 33, 40 and 47), the mice were sacrificed on day 55. The challenge and treatment protocol is shown in Fig. 2a, and the experiment was performed twice.

To identify the mechanism responsible for the beneficial effects of Hypo-EVs, another twelve mice were randomly divided into two groups (NC-Hypo-EVs, miR146a^KD^-Hypo-EVs). Each mouse was sensitized and challenged with OVA as described above, and at days 26, 33, 40, and 47, mice were injected with NC-Hypo-EVs (40 μg suspended in 0.1 mL PBS; intravenous) or miR146a^KD^-Hypo-EVs (40 μg suspended in 0.1 mL PBS; intravenous), respectively. Mice were sacrificed on day 55.

### EVs labeling and tracking in mice

Nor-EVs or Hypo-EVs were labeled with Dir (Invitrogen) as in our previous report [23]. For tracking the distribution of EVs, 100 μL PBS (as blank control), Dir-labeled Nor-EVs (40 μg suspended in 100 μL PBS), or Dir-labeled Hypo-EVs (40 μg suspended in 100 μL PBS) were injected into OVA-induced mice (day 26) via the tail vein. The mice were sacrificed on day 28, and lung fluorescence intensity was determined using an IVIS Spectrum (PerkinElmer, Waltham, MA) according to the manufacturer’s protocol.

### Analysis of cells and inflammatory cytokines in bronchoalveolar lavage fluid (BALF)

BALF was collected as previously described [22]. Briefly, left bronchial tubes of the mice were ligated, and the ice-cold PBS (0.5 mL) was instilled twice into the right lungs. Then, the collected BALF was centrifuged to pellet the cells and the supernatant was kept at − 80 °C until it was used for cytokine analysis. Cell pellets were resuspended in PBS (1 mL), and total quantity of inflammatory cells was counted using a hemocytometer, and eosinophils count was performed using Wright and Giemsa staining (BASO, Zhuhai, China).

Levels of IL-4 and IL-13 in the BALF were measured using commercial enzyme-linked immune sorbent assay (ELISA) according to the manufacturer’s instructions (R&D Systems, Minneapolis, MN).

### Lung histopathology

After BALF collection, the left lung tissues were fixed in 10% neutral-buffered formalin (48 h) and embedded in paraffin fixation. Then, the paraffin-embedded sections (4 μm thick, 3 sections per animal, 6 animals for each group) were stained with hematoxylin and eosin (H&E), periodic acid-Schiff (PAS), and Masson trichrome to evaluate the lung inflammatory or fibers level.

Inflammation score in the lungs were performed in a blind-way [24], inflammation was graded as follows: grade 0 (no inflammatory cells were observed), grade 1 (inflammatory cells were occasionally observed), grade 2 (bronchi were surrounded by 1–3 layer of inflammatory cells), grade 3 (bronchi or vessels were surrounded by 4–5 layer of inflammatory cells), and grade 4 (most bronchi or vessels were surrounded by more than 5 layer of inflammatory cells). Goblet cell hyperplasia was evaluated using the method described by Padrid et al. [25]. The pathological changes were quantified according to a modified five-point scoring system (grades 0–4) based on the percentage of goblet cells in the epithelium: grade 0 (no goblet cells), grade 1 (< 25%), grade 2 (25–50%), grade 3 (51–75%), and grade 4 (> 75%). At least 8 bronchioles were counted in each slide and then the mean inflammation score or the mean goblet cell hyperplasia score was calculated for each mouse.

Masson trichrome staining was employed to assess the collagen deposition in airways and lung parenchyma. The staining area (blue) in each paraffin-embedded slide was outlined using a light Axiovert 200 M microscope (Carl Zeiss GmbH, Jena, Germany); then, Image-Pro Plus software (Version X; Adobe, San Jose, CA) was used to quantify the areas occupied by collagen (blue), which were then divided by the total area examined (as the percentage of collagen fibers) [7]. At least 8 bronchioles or lung parenchyma were counted in each slide, the mean percentage of collagen fibers was calculated for each mouse.

The collagen content of the lung, determined as hydroxyproline content, was measured using a colorimetric assay (Nanjing Jiancheng Bioengineering Institute, Nanjing, China) according to the manufacturer’s protocol.

### Cell transformation

HLF-1 cells were seeded in 6-well plates (5 × 10^4^ cells per well), and divided into four groups: control, TGF-β1, TGF-β1+Nor-EVs, and TGF-β1+Hypo-EVs. After culturing overnight, the media were replaced with DMEM containing 2% EVs-free serum, and the cells were treated with Nor-EVs/Hypo-EVs (20 μg/mL) and/or TGF-β1 (10 ng/mL; Pepro Tech, Rocky Hill, NJ) for 48 h (HLF-1 cell preparations) and then harvested for protein or RNA extraction.

### Western blotting

The proteins of Nor-EVs, Hypo-EVs, lung tissues in mice, and HLF-1 cell preparations were extracted and quantified using a BCA Protein Assay Kit (Beyotime). The equal amounts of proteins (50 μg) were electrophoresed in 10% sodium dodecyl SDS-PAGE and transferred onto polyvinylidene difluoride membranes (Bio-Rad, Hercules, CA) pre-soaked with 100% methanol. Non-specific binding was blocked in Tris-buffered saline/0.05% Tween-20 (TBST) containing 5% non-fat milk powder, followed by incubation with the primary antibodies as in our previous description [26]. The antibodies of TSG101 (Cat#ab133586), HSP70 (Cat#ab181606), collagen-1 (Cat#ab260043), α-SMA (Cat#ab7817), TGF-β1 (Cat#ab215715), HIF-1α (Cat#ab179483), Gapdh (Cat#ab8245), β-actin (Cat#ab8226) were all purchased from Abcam (Cambridge, MA). Phospho-Smad2 (Ser465/467) + Smad3 (Ser423/425) rabbit polyclonal antibody (Cat#AF5920) was purchased from Beyotime. Then, the blots were washed with TBST and incubated with HRP-linked anti-rabbit/mouse IgG (Cat#ab97051/Cat#ab6728; Abcam) for 1 h at room temperature. Finally, immunoblot signals were visualized using ECL chemiluminescence kit (Millipore, Billerica, MA) and imaged using a ChemiScope 3400 Mini (CLINX Science Instruments, Shanghai, China). For the detection of EV biomarkers (HSP70 and TSG101), Ponceau S staining served as a loading control [27]. Apart from HSP70 and TSG101, other band intensities were quantitated using Image J software (National Institutes of Health, Bethesda, MD), and data were normalized against that of the corresponding GAPDH bands (loading control). Results are reported as fold increase over the control group.

### RNA isolation and quantitative real-time PCR

RNA was extracted from the Nor-MSCs, Hypo-MSCs, Nor-EVs, Hypo-EVs, lung tissues, and HLF-1 cell preparations using mirVana RNA isolation kit (Ambion, Austin, TX) according to the manufacturer’s manual. All of the primers for real-time PCR (miR-146a-5p, let-7, miR-485, miR-29b, miR-30a and U48) were purchased from Genecopoeia (Germantown, MD). Real-time PCR was performed with All-in-one™ qPCR Mix (Genecopoeia) in a CFX96™ Real-Time system (Bio-Rad, Hercules, CA). The relative expression of miRNA was normalized to U48 and evaluated by the 2^−ΔΔCt^ method, based on our previous description [26].

### Statistical analysis

The statistical analyses were performed with GraphPad Prism (Version 5.0; La Jolla, CA). Data are expressed as mean ± SD. The groups were compared using the Student’s *t* test, one-way analysis of variance (Tukey-Kramer post hoc tests). Data were considered statistically significant for *P* values less than 0.05.

## Results

### Isolation and characterization of EVs derived from hUCMSCs under hypoxic conditions

**Fig. 1:**
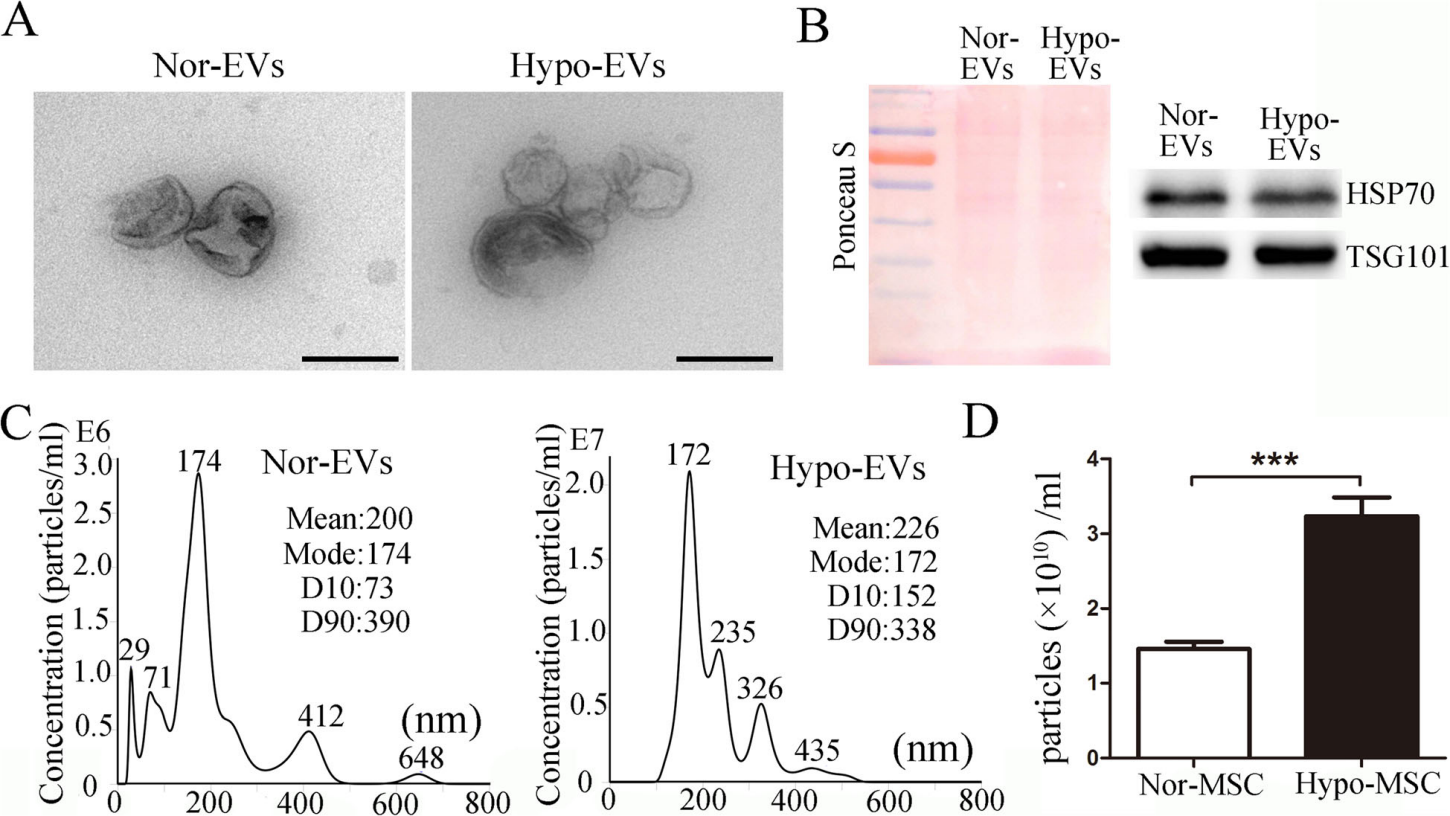
Characterization of Nor-EVs and Hypo-EVs. **a** Transmission electron micrographs of Nor-EVs and Hypo-EVs. Scale bar = 200 nm. **b** Western blot analysis of TSG101 and HSP60 expression in Nor-EVs and Hypo-EVs (*n* = 3). Ponceau S staining served as a loading control. **c** NTA analysis of Nor-EVs and Hypo-EVs revealed that EVs from the two groups exhibit similar size ranges (*n* = 3). **d** NTA counted the production of EVs derived from normoxia-cultured hUCMSCs (Nor-MSC) and EVs from hypoxia-treated hUCMSCs (Hypo-MSC) (*n* = 3). ***P < 0.001

HUCMSCs were prepared as in our previous report [19] and seeded for 24 h under one of the following two culture conditions: normoxic (21% O_2_; Nor-MSC) and hypoxic preconditioning (5% O_2_; Hypo-MSC). Cell proliferation of Nor-MSC and Hypo-MSC was evaluated at the 24 h, using a BrdU-uptake assay. Results showed that the proliferation was obviously increased in hypoxic group. In addition, we found that the viability of hUCMSCs was also increased at 24 h of hypoxic exposure (see Additional file 1). EVs were gathered from Nor-MSC or Hypo-MSC conditioned medium separately by ultracentrifugation and named Nor-EVs and Hypo-EVs. Then, Nor-EVs and Hypo-EVs were characterized by using transmission electron microscope (TEM), Western blot, and nanoparticle tracking analysis (NTA). As shown in Fig. 1a, TEM showed typically round nanoparticles, which have diameters of about 200 nm. No morphological difference between the two kinds of EVs was observed with regard to their shape. Western blot revealed that several EV markers including tumor susceptibility gene 101 (TSG101) and heat shock protein 70 (HSP70) [28] were detected in these EVs (Fig. 1b). NTA exhibited that the mean sizes of Nor-EVs and Hypo-EVs were 150 ± 43 and 160 ± 57 nm, respectively (Fig. 1c). This result indicated a slight difference in size between the two conditions, and this difference was not significant. In addition, we found that exposure of hUCMSCs to hypoxia significantly increased the total EV particles (Nor-EVs, (1.45 ± 0.1) × 10^10^ particles/mL; Hypo-EVs, (3.23 ± 0.25) × 10^10^ particles/mL) as shown in Fig. 1d, suggesting that hypoxic conditioning induced an increased release of EVs when compared with the normoxic control.

### Administration of Hypo-EVs attenuated OVA-induced chronic airway inflammation in mice

**Fig. 2:**
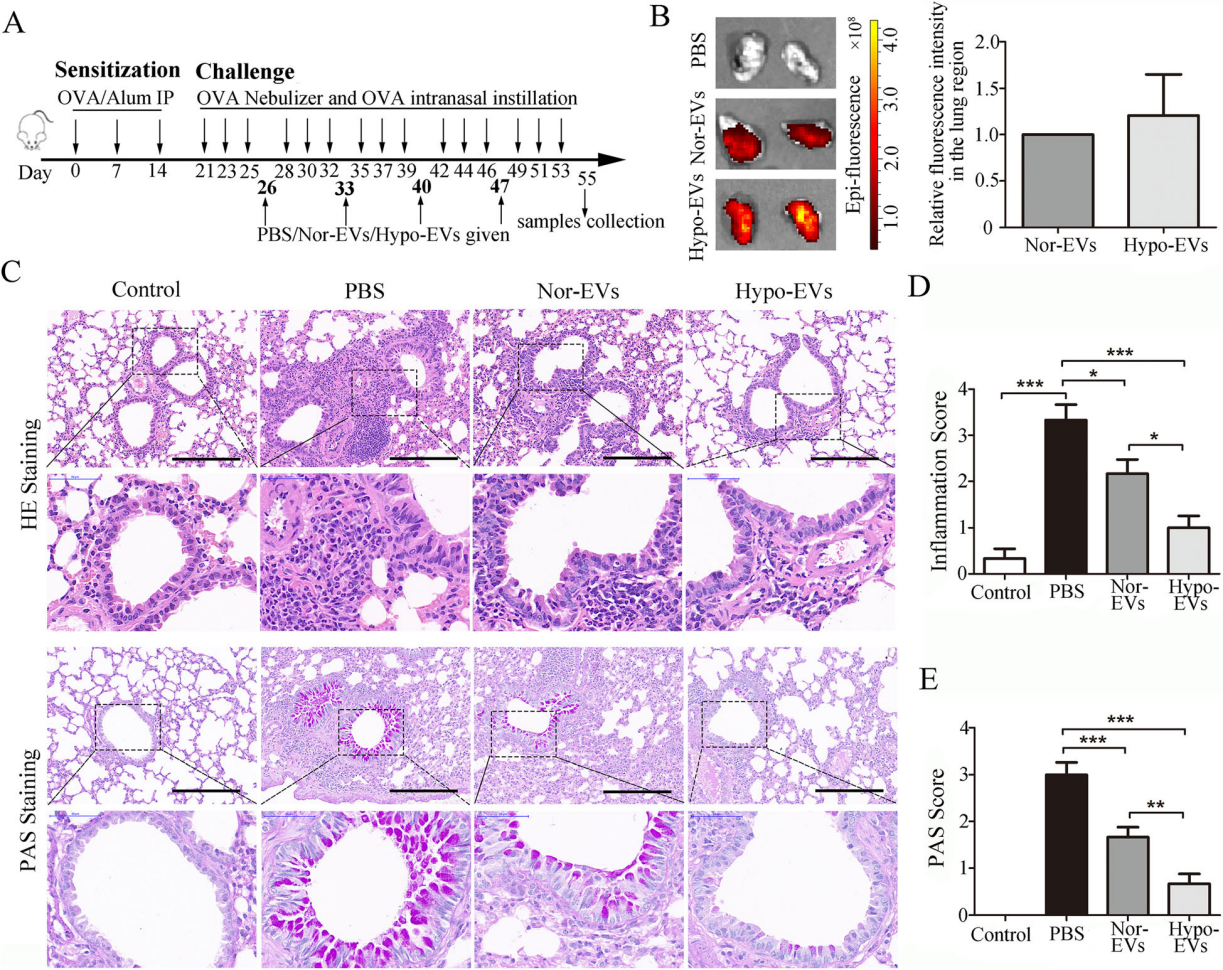
Administration of Hypo-EVs attenuated OVA-induced chronic airway inflammation in mice. **a** Schematic illustration of the establishment of mouse chronic allergic airway inflammation model. **b** Distribution of DIR-labeled EVs in OVA-induced mice lung after tail vein administration (26 day injection and 28 day detected). Representative ex vivo bioluminescence images of mice lungs from different groups (left), and the quantification of the relative fluorescence intensity in the lungs (right; *n* = 3 from three independent experiments). **c** Representative photographs of HE- and PAS-stained lung sections from each group (black bar = 200 μm). **d**, **e** The inflammatory infiltration and goblet cell hyperplasia were quantified by HE and PAS scores (*n* = 6). *P < 0.05, **P < 0.01, ***P < 0.001

To test the therapeutic effect of Hypo-EVs, the asthmatic mouse model was performed according to the previous method [20, 21]. Hypo-EVs were intravenously transplanted into mice at the time of the challenge phase every week (a total of four times), whereas Nor-EVs and PBS were used as controls (Fig. 2a). First, we used DiR-labeled EVs to detect the distribution of Nor-EVs or Hypo-EVs in asthma mice and found that both of the EVs could be delivered into the mice lung region, and the fluorescence intensities of the lung region between the two conditions were not significantly different (Fig. 2b and Additional file 2). HE and PAS staining showed that the OVA-challenged mice treated with PBS (PBS group) presented abundant infiltrates of peribronchial and perivascular inflammatory cells and obvious mucus production in epithelia layers. Compared to PBS group, treatment with Nor-EVs or Hypo-EVs significantly attenuated the above allergic inflammation and inhibited mucus secretion; moreover, the inflammatory and PAS score of the mice injected with Hypo-EVs was much lower (53% and 60% decrease, respectively) than that in the mice of the Nor-EV group (Fig. 2c–e). These data suggest that Hypo-EVs are able to attenuate chronic airway inflammation.

### Hypo-EV administration reduced inflammatory cell infiltration and inflammatory cytokine levels in the bronchoalveolar lavage fluid (BALF)

**Fig. 3:**
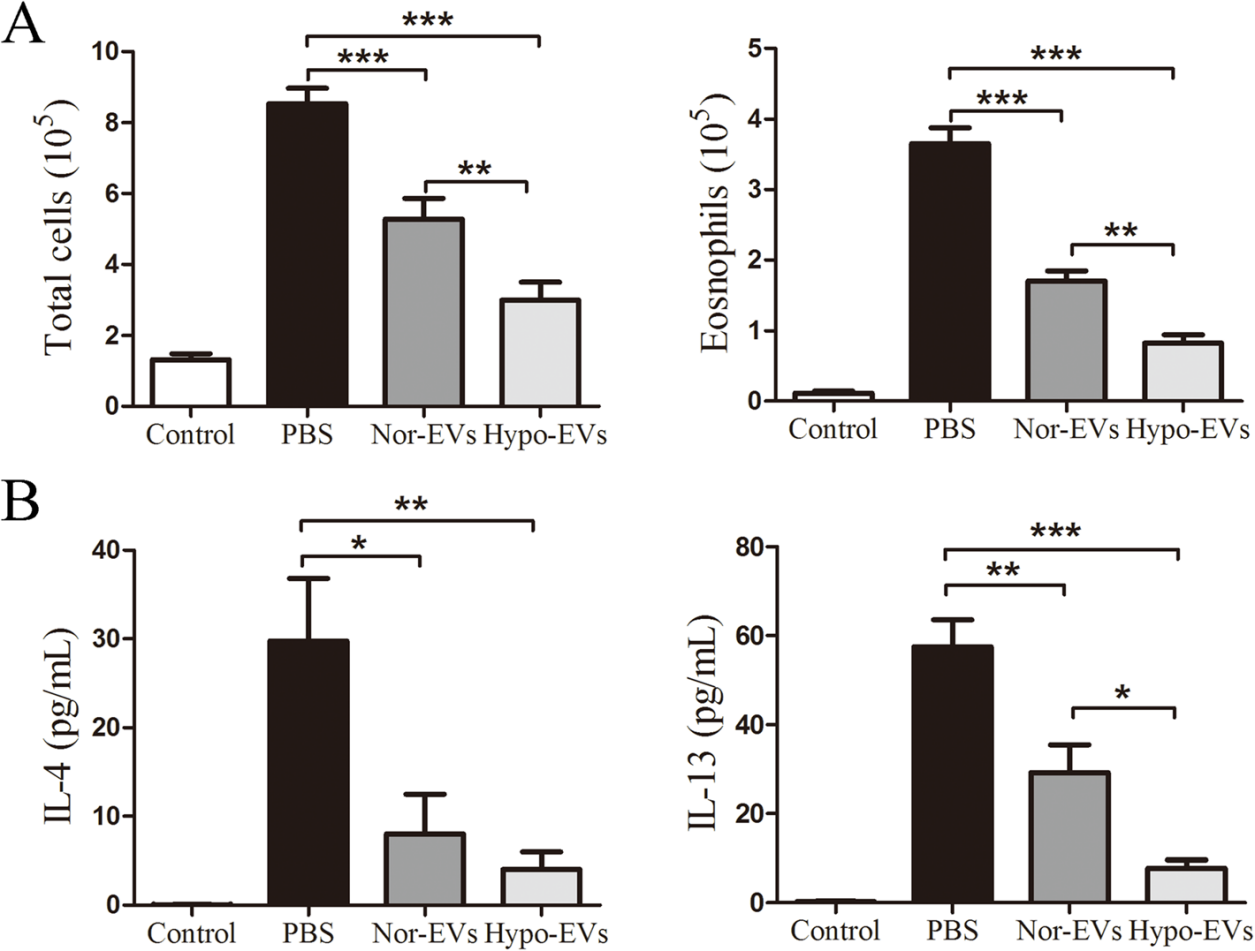
Hypo-EV treatment reduced inflammatory cell infiltration and inflammatory cytokines in the BALF. **a** Statistical analysis of the total inflammatory cells (left) and eosinophils (right) in the BALF (*n* = 6). **b** Statistical analysis of IL-4 (left) and IL-13 (right) levels in the BALF as measured by ELISA (*n* = 6). *P < 0.05, **P < 0.01, ***P < 0.001

The effects of Hypo-EVs on the airway inflammatory cells in the BALF from OVA-induced asthmatic mice were further determined. Compared to the control group, the numbers of total inflammatory cells and eosinophils in the PBS group were increased. Hypo-EV administration dramatically decreased both total inflammatory cells and eosinophil numbers in the BALF, compared with the PBS even or Nor-EV treatments (Fig. 3a). Allergic airway inflammation is mainly accompanied by the release of pro-inflammatory type 2 cytokines [29]. Thus, the levels of the IL-4 and IL-13 in the BALF after administration of Hypo-EVs were examined using ELISA assay. Similar with the previous reports [21, 30, 31], the concentrations of IL-4 and IL-13 in the control group were extremely lower, and compared to that, increased levels of IL-4 and IL-13 from PBS group were observed. Treatment with Nor-EVs or Hypo-EVs was able to significantly decrease the levels of IL-4 and IL-13. In addition, compared to the OVA-challenged mice treated by Nor-EVs, injection of Hypo-EVs observably decrease the IL-13 levels (73% decrease), and no significant differences in IL-4 levels were found among these two EV groups (Fig. 3b). These findings demonstrate that Hypo-EVs are able to attenuate inflammatory cell and type 2 cytokine infiltration in the OVA-induced mouse lung.

### Hypo-EV administration prevented lung remodeling in chronic OVA mice

**Fig. 4:**
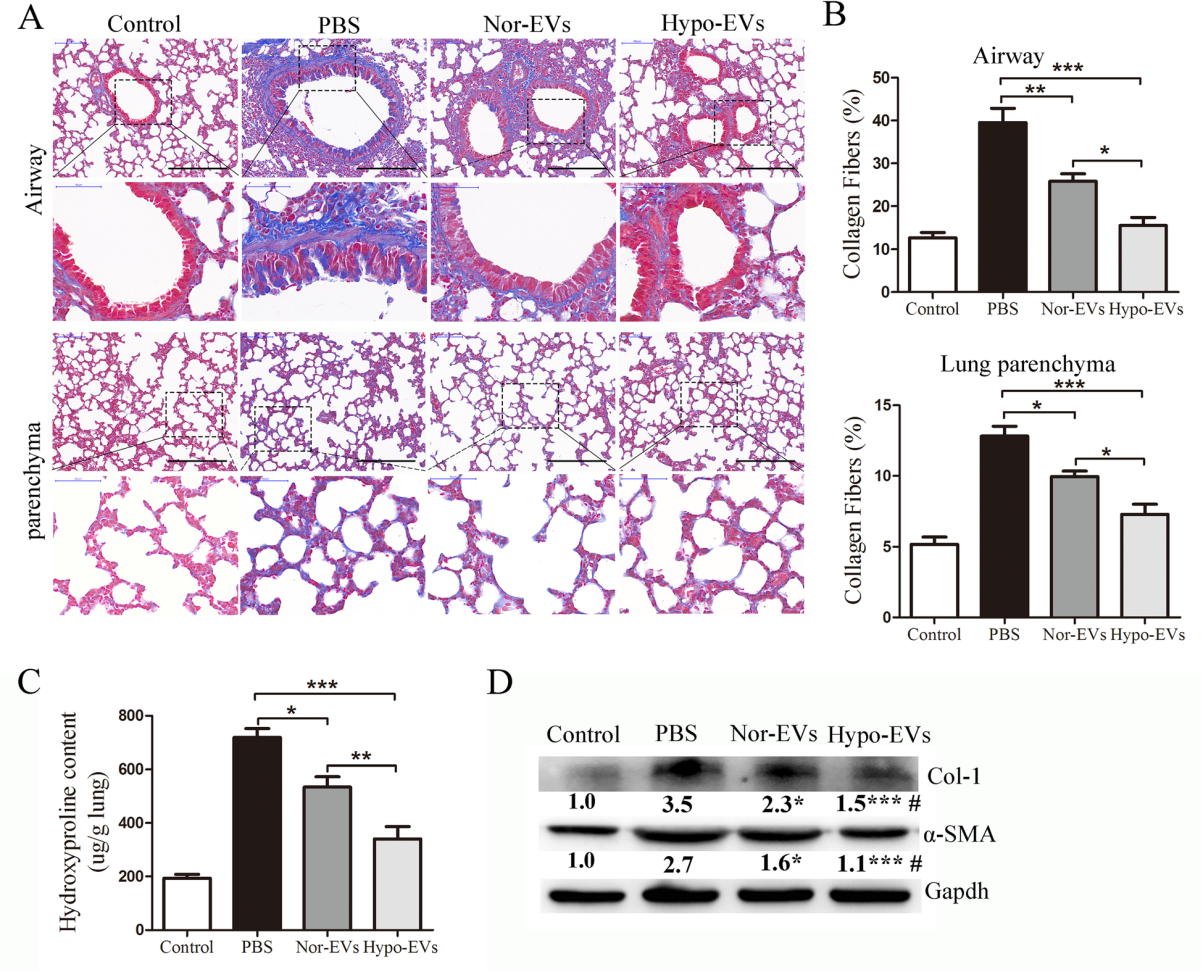
Hypo-EV treatment prevented the airway and lung parenchyma fibrosis in chronic asthma mice. **a** Representative photomicrographs of airway (upper panels) and lung parenchyma (lower panels) stained with Masson trichrome staining (black bar = 200 μm). **b** Percentage of collagen fiber content in airway and lung parenchyma (*n* = 6). *P < 0.05, **P < 0.01, ***P < 0.001. **c** Collagen levels in lung tissue determined by hydroxyproline assay (*n* = 6). *P < 0.05, **P < 0.01, ***P < 0.001. **d** Representative Western blots of Col-1 and α-SMA (GAPDH was used as protein loading control) in the lungs from each group (*n* = 4), and band intensities were normalized against the corresponding GAPDH and the numbers are presented as fold increase over the control group. *P < 0.05, ***P < 0.01 vs. PBS group; #*P* < 0.05 vs. Nor-EV group

A recent study showed that MSC-EVs can significantly inhibit bronchial subepithelial and pulmonary fibrosis in chronic asthma mice [7]. We thus assessed whether Hypo-EVs have a similar or better effects on lung remodeling in asthma. In PBS group animals, collagen fiber content increased dramatically both in lung parenchyma and airways compared to the control group, as shown by Masson trichrome staining (Fig. 4a, b). Nor-EV and Hypo-EV treatment resulted in a significant decrease in collagen formation, and compared with Nor-EV administration, Hypo-EVs markedly decreased collagen fiber deposition in lung airways (39% decrease) and parenchyma (26% decrease) (Fig. 4a, b). Similarly, hydroxyl proline assay of total lung collagen showed that Hypo-EVs further suppressed OVA-challenge-induced hydroxyl proline levels (36% decrease) compared with Nor-EV treatment (Fig. 4c). Less collagen-1 was detected in the lung tissues of Hypo-EV-treated mice compared with that in the lungs of Nor-EV mice (Fig. 4d).

Collagen-1 is mainly produced by α-SMA expressing cells. Therefore, we further investigated the expression of α-SMA in lung tissues and found that Hypo-EVs significantly inhibited the expression of α-SMA compared with that in the Nor-EV group (Fig. 4d). These data suggest that Hypo-EV treatment is effective in preventing airway remodeling in chronic airway inflammation.

### Hypo-EV administration inhibited TGF-β1-Smad2/3 signaling pathway

**Fig. 5:**
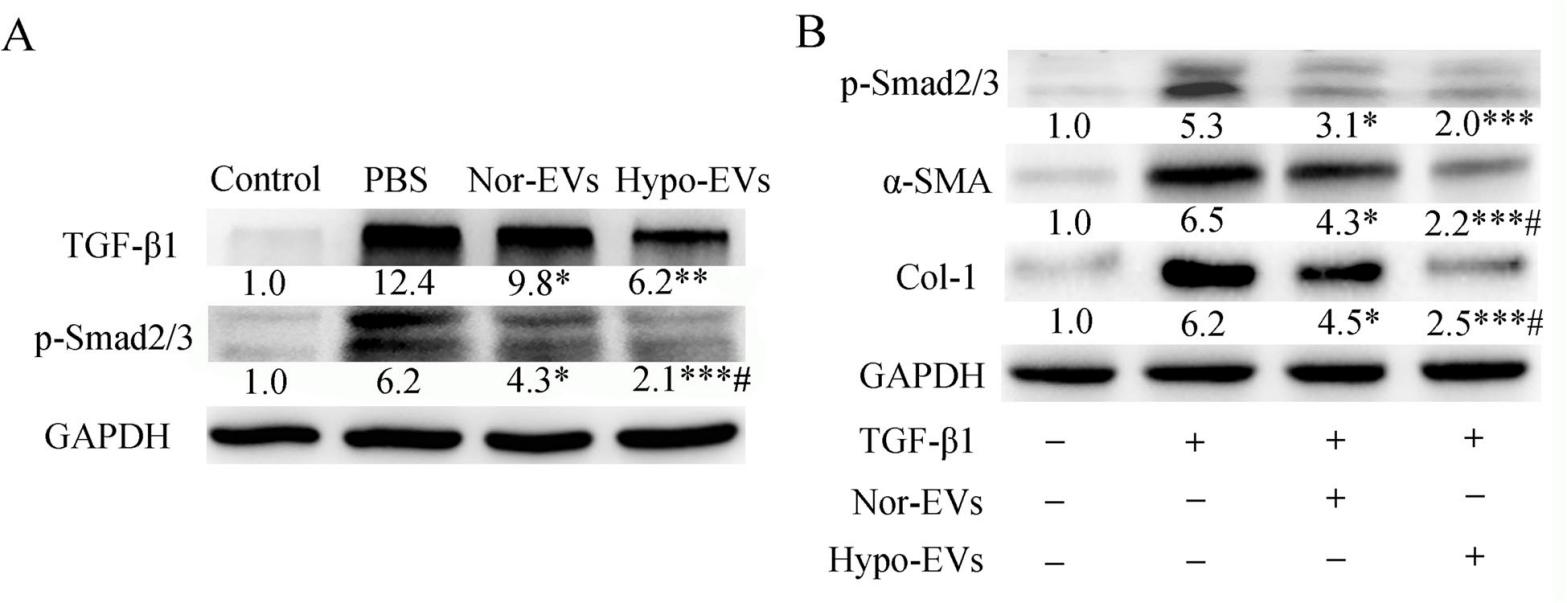
Effect of Hypo-EVs on the expression of TGF-β1-phosphorylated (p)-Smad2/3 in lung tissues and in TGF-β1-treated HLF-1 cells. **a** Representative Western blots of TGF-β1 and p-Smad2/3 (GAPDH was used as protein loading control) in the lungs from each group (*n* = 4), and band intensities were normalized against the corresponding GAPDH and the numbers are presented as fold increase over the control group. *P < 0.05, **P < 0.01, ***P < 0.001 vs. PBS group; ^#^*P* < 0.05 vs. Nor-EV group. **b** The expression of p-Smad2/3, α-SMA, collogen-1, and GAPDH proteins in TGF-β1-treated HLF-1 cells were determined by Western blotting (*n* = 3 from three independent experiments). Band intensities were normalized against the corresponding GAPDH (protein loading control) and the numbers are presented as fold increase over the control group. *P < 0.05, ***P < 0.001 vs. TGF-β1 group; #*P* < 0.05 vs. TGF-β1+Nor-EV group

TGF-β1 signaling is one of the main factors that induce α-SMA expression [32]. In our previous study, we found that Nor-EVs could significantly inhibit the TGF-β1-mediated upexpression of α-SMA [23]. Therefore, we further evaluated the effect of Hypo-EVs in TGF-β1 signaling. Western blot analysis revealed that Nor-EV or Hypo-EV administration significantly reduced the levels of TGF-β1 and p-Smad2/3 in the OVA mice lung tissues compared with PBS treatment. Furthermore, compared with the Nor-EV administration, Hypo-EV treatment dramatically decreased p-Smad2/3 expression (Fig. 5a). To confirm the effects of Hypo-EVs on the regulation of TGF-β1-induced α-SMA, we conducted an in vitro study by using a human lung fibroblast cell line (HLF-1) stimulated by TGF-β1 [33]. As seen in Fig. 5b, Nor-EVs or Hypo-EVs remarkably mitigated TGF-β1-induced elevation of p-smad2/3, α-SMA, and collgen-1 in HLF-1 cells. Moreover, the protein expression of α-SMA and collgen-1 in TGF-β1-treated HLF-1 cells significantly reduced under exposure to Hypo-EVs compared with that after exposure to Nor-EVs. Taken together, these results indicate that Hypo-EVs suppress HLF-1 cells activation induced by TGF-β1 and inhibited TGF-β1-Smad2/3 signaling pathway in OVA mice.

### Decreased miR-146a-5p expression impaired hypo-EV-mediated lung protection

**Fig. 6:**
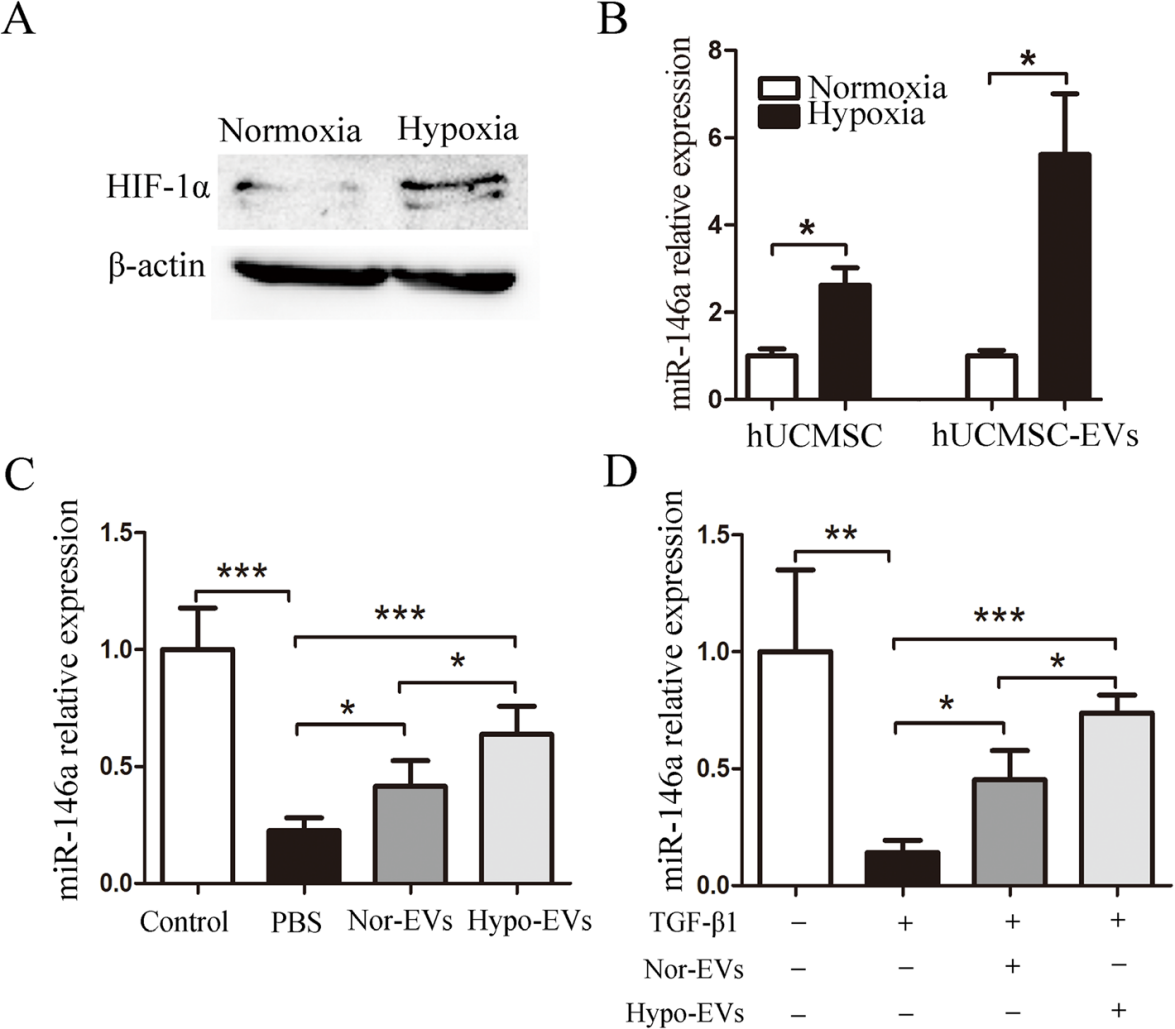
The expression of miR-146a-5p in Hypo-EVs. **a** The protein expression of HIF-1α in hUCMSCs under normoxic and hypoxic conditions. **b** The expression of miR-146a-5p in hUCMSCs or EVs were detected by real-time PCR (*n* = 3). **c** MiR-146a-5p expression in the lung tissues of the control, PBS, Nor-EV, and Hypo-EV group (*n* = 6). **d** The expression of miR-146a-5p in HLF-1 cells that were treated with Nor-EVs or Hypo-EVs were detected by real-time PCR (*n* = 3). *P < 0.05, **P < 0.01, ***P < 0.001

It has been reported that hypoxia culture enhances the anti-inflammatory potency of MSCs in OVA mouse, and miR-146a-5p is critical for these properties [18]. Apart from modulating anti-inflammatory responses [34, 35], miR-146a-5p also has the effect of inhibiting TGF-β1-Smad signaling [36]. After hUCMSCs were exposed to hypoxia for 24 h, the expression of HIF-1α was increased compared with that of hUCMSC cultured in normoxia (Fig. 6a). In addition, the miR-146a-5p expression in Hypo-MSC or Hypo-EVs was significantly increased compared with that in Nor-MSC or Nor-EVs, respectively (Fig. 6b). We also screened Nor-EVs or Hypo-EVs for several known anti-inflammatory and anti-remodeling miRNAs including let-7, miR-485, miR-29b, and miR-30a [37], which are regulated under hypoxia in MSCs [18, 38]. Intriguingly, among the miRNAs, miR-146a-5p was the most abundant in Nor-EVs or in Hypo-EVs (see Additional file 3). Hypo-EV or Nor-EV treatment significantly increased the miR-146a-5p expression in OVA mice lung tissues in vivo and TGF-β1-treated HLF-1 in vitro; moreover, compared with the Nor-EVs group, Hypo-EV administration dramatically increased the expression of miR-146a-5p in the lung (1.5 fold increase) or HLF-1 cells(1.6 fold increase) (Fig. 6c, d).

**Fig. 7:**
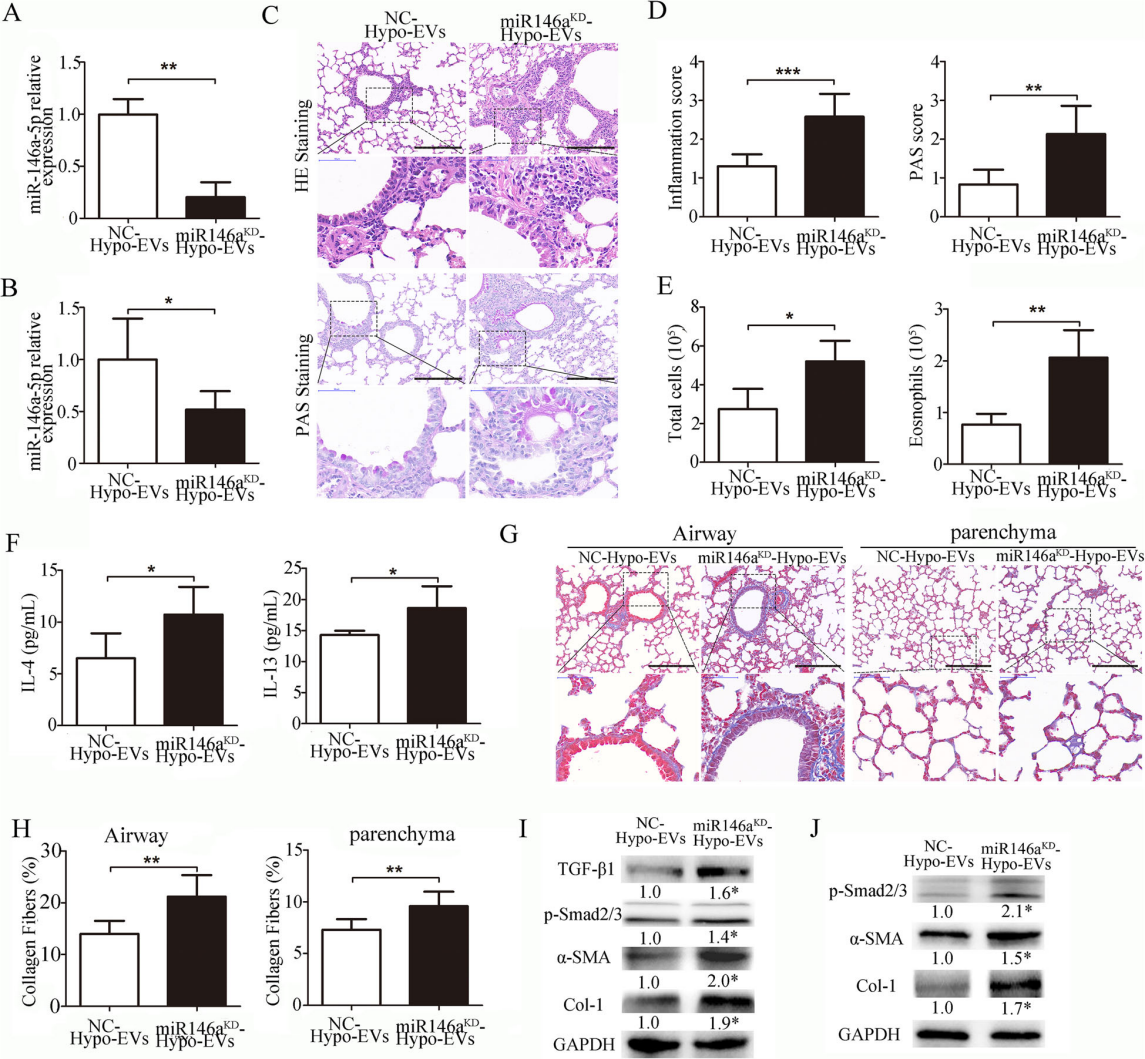
Inhibition of miR-146a-5p in Hypo-EVs impaired Hypo-EV-mediated lung protection in a mice model of asthma. **a** miR-146a-5p expression was detected by real-time PCR in EVs isolated from the hypoxic medium of hUCMSCs treated with 100 nM inhibitor-146a-5p or negative control (NC) (*n* = 3). **b** Expression of miR-145a-5p in lung tissue after injection of miR146a^KD^-Hypo-EVs or NC-Hypo-EVs into the asthma mice (*n* = 6). **c** Representative photographs of HE- and PAS-stained lung sections from each group (black bar = 200 μm). **d** The inflammatory infiltration and goblet cell hyperplasia were quantified by HE and PAS scores (*n* = 6). **e** Statistical analysis of the total inflammatory cells and eosinophils in the BALF (*n* = 6). **f** Statistical analysis of IL-4 and IL-13 levels in the BALF as measured by ELISA (*n* = 6). **g** Representative photomicrographs of airway and parenchyma stained with Masson trichrome staining (black bar = 200 μm). **h** Percentage of collagen fiber content in airway and lung parenchyma (*n* = 6). **i** Representative Western blots of TGF-β1, p-Smad2/3, α-SMA, and collogen-1 in the lungs from each group (*n* = 3), and band intensities were normalized against the corresponding GAPDH and the numbers are presented as fold increase over the NC-Hypo-EV group. **j** The expression of p-Smad2/3, α-SMA, and collogen-1 in TGF-β1-treated HLF-1 cells were determined by Western blotting (*n* = 3). Band intensities were normalized against the corresponding GAPDH and the numbers are presented as fold increase over the NC-Hypo-EV group. *P < 0.05, **P < 0.01, ***P < 0.001

To identify whether miR-146a-5p is responsible for the beneficial effects of Hypo-EVs, the miR-146a-5p expression in Hypo-EVs was knocked down (called miR146a^KD^-Hypo-EVs) via miR-146a-5p silencing (Fig. 7a). Then, miR146a^KD^-Hypo-EVs or their control NC-Hypo-EVs were injected into mice with OVA-induced asthma. Our results showed that miR146a^KD^-Hypo-EV treatment resulted in a decreased miR-146a-5p expression in the lungs compared with that in the NC-Hypo-EVs-treated mice (Fig. 7b), partly suggesting that a direct transfer of miR-146a-5p is one of the reasons for the Hypo-EV-mediated upregulation of miR-146a-5p. Results revealed that the degree of increase in the inflammation and PAS score, the number of total cells and eosinophils, the content of IL-4 and IL-13, the area of collagen fibers, and the expression of TGF-β1-Smad2/3 signaling in miR146a^KD^-Hypo-EV-treated mice was higher than those in NC-Hypo-EV-treated mice (Fig. 7c–i). miR146a^KD^-Hypo-EVs or NC-Hypo-EVs were administered to TGF-β1-treated HFL-1 cells in vitro. As shown in Fig. 7j, miR146a^KD^-Hypo-EVs significantly increased the protein expression levels of p-smad2/3, a-SMA, and collagen-1 compared with those of NC-Hypo-EVs. These findings demonstrated that miR-146a-5p knockdown impaired Hypo-EV-mediated lung protection in mice with OVA-induced asthma.

## Discussion

An ideal treatment for asthma would effectively act not only on inflammation but also on airway remodeling. Although MSCs and its substitute MSC-EVs have shown good anti-asthma effects [4, 5, 7, 10–13], how to further energize their potential therapeutic outcomes is of great interest. Recently, Kwak et al. [18] found that pretreatment of transplanted human umbilical cord blood-derived MSCs with CoCl_2_ (hypoxia-mimetic reagent) can suppress lung inflammation more than naïve MSCs can in a mouse model of asthma. In this study, we further demonstrated that compared with normoxic hUCMSC-EVs, hypoxic hUCMSC-EVs exhibit enhanced anti-inflammation and anti-fibrosis potential, thus possibly being a better choice for the treatment of asthma.

With their higher accessibility, higher cell vitality, lower senescence, and fewer ethical constraints than other adult counterparts, hUCMSCs are viewed as a better choice of MSCs for clinical application [39], and banks of hUCMSCs are being set up in many countries [40], which is why we selected hUCMSCs as the source of stem cell-derived EVs. The physiological oxygen tension in the umbilical cord is about 5% [41], and similar to a previous study [42], the cell proliferation and viability of MSCs was increased at 24 h of hypoxic exposure, which might be an explanation for the more release of EVs under hypoxia. Our data revealed that hypoxia could not alter the average size of EVs. By contrast, Han et al. [43] showed that hypoxia treatment (5%O_2_, 48 h) promoted adipose MSCs to produce larger EVs (Nor-EVs 75 nm versus Hypo-EVs 130 nm). These discrepancies are likely due to the different MSC types, different culture media composition, and different hypoxia treatment timing.

In recent years, the behavior of hypoxic MSC-EVs from different sources has been investigated, and hypoxia preconditioning has been found to increase the therapeutic potential of MSC-EVs in the treatment of myocardial infarction [17], Alzheimer’s disease [44], acute kidney disease [45], and traumatic spinal cord injury [46]. However, to date, no study had evaluated the therapeutic administration of Hypo-EVs in experimental allergic asthma. A recent study has shown that a systemic dose of about 40 μg Nor-EVs per mouse is effective in experimental asthma [7]. In this study, we first revealed that Nor-EVs or Hypo-EVs are able to inhabit the lung site in OVA-induced asthma mice, indicating that Hypo-EVs are capable of performing functions within an injured lung. Then, we used an equivalent and repeated dose of Hypo-EVs (40 μg per mouse; four times) and found that Hypo-EV administration is more potent than Nor-EVs in terms of alleviating inflammatory cell infiltration, goblet cell hyperplasia, mucus production, and lung fibrosis in asthmatic mice. These findings contribute to previous results that MSC-EVs constitute a therapeutic alternative to the usually tested MSC-based approaches [9]. They also provide that hypoxic hUCMSC-EVs may offer a desirable therapeutic strategy for chronic asthma prevention in comparison with normoxic hUCMSC-EVs. However, Hypo-EV should be cautiously used under malignant conditions, as EVs secreted by hypoxia pre-challenged MSCs promote the growth and mobility of non-small cell lung cancer cells [47].

The chronic airway inflammation of asthma is characterized by the release of type 2 T helper cell cytokines, such as IL-4 and IL-13 [29]. These cytokines induce eosinophil recruitment, airway hyperresponsiveness, epithelial cell apoptosis, goblet cell hyperplasia, mucus production, fibroblast proliferation, and extracellular matrix deposition [29, 48]. Obviously, the consistent inflammation in chronic asthma leads to airway remodeling. Importantly, our results demonstrated that Hypo-EVs could significantly downregulate the levels of IL-4 and IL-13 in BALF. As such, inflammation was likely attenuated, and fibrosis was possibly prevented with Hypo-EV treatment.

TGF-β1 is also believed to play an important role in inflammatory processes. It exerts immunosuppressive proprieties by regulating lymphocyte homeostasis, inhibiting Th1 and Th2 cell responses, and promoting the differentiation of Treg. Also, it displays pro-inflammatory roles by inducing the differentiation of T lymphocytes into Th17, which then amplifies the inflammatory component. Besides, TGF-β1 is a potent chemotactic factor that promotes the rapid infiltration and accumulation of inflammatory cells, indicating the complexity of TGF-β1 in inflammatory regulation [49, 50]. Apart from immunomodulatory functions, TGF-β1 participates in extracellular matrix synthesis and airway remodeling that characterizes chronic asthma [51]. The stimulation by TGF-β1 induces the phosphorylation of the downstream targets Smad2/Smad3 (p-Smad2/3); then, p-Smad2/3 will be transported to the nucleus, stimulating fibroblasts to become collagen-producing myofibroblasts (especially increasing α-SMA expression) [52]. Thus, therapeutic approaches modulating the TGF-β1 signaling pathway may target fibrosis in asthma [49]. Our group and other researchers previously reported that MSC-EVs have the ability to reduce the expression of α-SMA and collagen-1 in liver sites in *S. japonicum* or CCL4 experimental mice, respectively [23, 53]. In this study, we found that the administration of Nor-EVs or Hypo-EVs could noticeably reduce the expression of collagen-1 and α-SMA, concomitant with a decrease of TGF-β1-pSmad2/3 signaling in OVA mice. Similar to our report, de Castro et al. [7] found that the TGF-β1 levels in OVA mice lung tissue were higher and reduced significantly by MSC-EVs. In the present study, we also showed that Hypo-EVs suppress TGF-β1-induced activation in HLF-1 cells in vitro, which suggested that Hypo-EVs may modulate airway remodeling via regulating the expression of signaling molecules of the TGF-β1-Smad pathway, while we did not rule out the possibility that the amelioration of lung fibrosis might be due to the direct anti-proliferative effect of Hypo-EVs on lung myofibroblasts.

In recent years, miRNAs have been shown to be an important beneficial mechanism in the immune microenvironment. Especially, it has been reported that miR-146a-5p could inhibit the proliferation and function (IL-13 secretion) of type II innate lymphoid cells (ILC2), efficiently protect mice against OVA-induced allergic asthma [34, 35], and also modulate anti-fibrosis responses induced by TGF-β1-Smad signaling [36]. The content of miR-146a-5p is relatively abundant in hUCMSC-EVs in accordance with a previous study [54]. In addition, its level dramatically increased in Hypo-EVs. Based on the expression of miR-146a-5p was significantly increased in the lung tissue treated with Hypo-EVs and that miR-146a-5p can be transferred into OVA mice lung tissue through Hypo-EVs, we hypothesized that Hypo-EVs may protect mice against OVA-induced allergic asthma through the transfer of miR-146a-5p. Indeed, we observed that decreased miR-146a-5p expression in Hypo-EVs impaired Hypo-EV-mediated lung protection in OVA mice, which supported our hypothesis. Specifically, Hypo-EVs might decrease IL-13 levels in BALF from the mice with OVA-induced asthma through miR-146a-5p delivery; however, by using TargetScan analysis (http://www.targetscan.org/vert_72/), we found that miR-146a-5p had no binding site (conserved sites) with the 3′ untranslated regions of IL-13 (data not shown), which indicated that miR-146a-5p could not directly act on IL-13. Thus, whether Hypo-EVs could affect IL-13 levels by regulating ILC2 remained unclear. Hypoxia preconditioning could be used as an efficient method to enrich miR-146a-5p in MSC-EVs, which indicates that Hypo-EVs may be used for the treatment of other miR-146a-5p abnormal diseases, such as inflammatory arthritis [55] and experimental autoimmune encephalomyelitis [56]. These matters should be addressed in the following study.

This study does have a number of limitations: (1) Due to the lack of relevant equipment, some pulmonary function data, such as airway hyperresponsiveness, dynamic or static compliance, were not detected. (2) Different concentrations of Hypo-EVs should be investigated in dose/response experiments, which are used to estimate the dosage of clinical medication. (3) Only IL-4 and IL-13 in BALF were analyzed in this experiment, a wider range of mediators (inflammatory cells and cytokines) should be detected to further elucidate the mechanisms of action of Hypo-EVs. (4) Whether other substances in Hypo-EVs, such as other miRNAs, proteins, and lipids, play roles in the Hypo-EVs-mediated lung protection in experimental allergic asthma remains to be investigated in future studies.

## Conclusions

We have shown that reduction of O_2_ oxygen tension increases EV secretion by hUCMSCs and enhances some aspects of their anti-inflammatory and anti-fibrosis properties, such as miR-146a-5p. These changes may result in improved outcomes for hUCMSC-EVs associated with inhibition of TGF-β1/Smad2/3 signaling and preservation of airway inflammation and fibrosis. These findings indicate that hypoxia may be an important tool in promoting the production of hUCMSC-EVs for the treatment of asthma diseases.

## Supplementary Information

**Additional file 1. **Characterization of hUCMSCs cultured under hypoxic condition (5% O_2_). hUCMSCs were subjected to hypoxia for 24 h, **A** cell proliferation was assessed by using a BrdU-uptake kit (*n* = 5), **B** cell viability was evaluated by CCK-8 assay (*n* = 8). *P < 0.05, ***P < 0.001.

**Additional file 2.** Biodistribution of DiR-labeled EVs in OVA-mice. Analysis of DiR-labeled EVs (PBS was used as a blank control) after systemic administration was detected using an in vivo imaging system.

**Additional file 3. **The expression of miR-146a-5p, let-7, miR-484, miR-29b, and miR-30a in Nor-EVs and Hypo-EVs. The expression levels of the miRNAs were normalized to U48 (*n* = 3). **P < 0.01.

## Data Availability

All data generated or analyzed during this study are included in this published article.

## Abbreviations

MSCs: Mesenchymal stem cells
hUCMSCs: Human umbilical cord MSCs
EVs: Extracellular vesicles
Nor-EVs: Normoxic hUCMSC-EVs
Hypo-EVs: Hypoxic hUCMSC-derived EVs
HSP70: Heat shock protein 70
TSG101: Tumor susceptibility gene 101
α-SMA: α-Smooth muscle actin
PBS: Phosphate-buffered saline
HLF-1: Human lung fibroblasts
HE: Hematoxylin and eosin
Masson: Masson trichrome staining
OVA: Ovalbumin
PAS: Periodic acid-Schiff
TGF-β1: Transforming growth factor-β1
BALF: Bronchoalveolar lavage fluid
IL: Interleukin
ELISA: Enzyme-linked immune sorbent assay
NTA: Nanoparticle trafficking analysis
TEM: Transmission electron microscope
HIF-1α: Hypoxia inducible factors 1α
CCL4: Carbon tetrachloride
