# Supplementary figures and images for: Hypoxic hUCMSC-derived extracellular vesicles attenuate allergic airway inflammation and airway remodeling in chronic asthma mice
Source: Stem Cell Res Ther. 2021 Jan 6;12:4. doi: 10.1186/s13287-020-02072-0 (PMC7789736; doi:10.1186/s13287-020-02072-0)

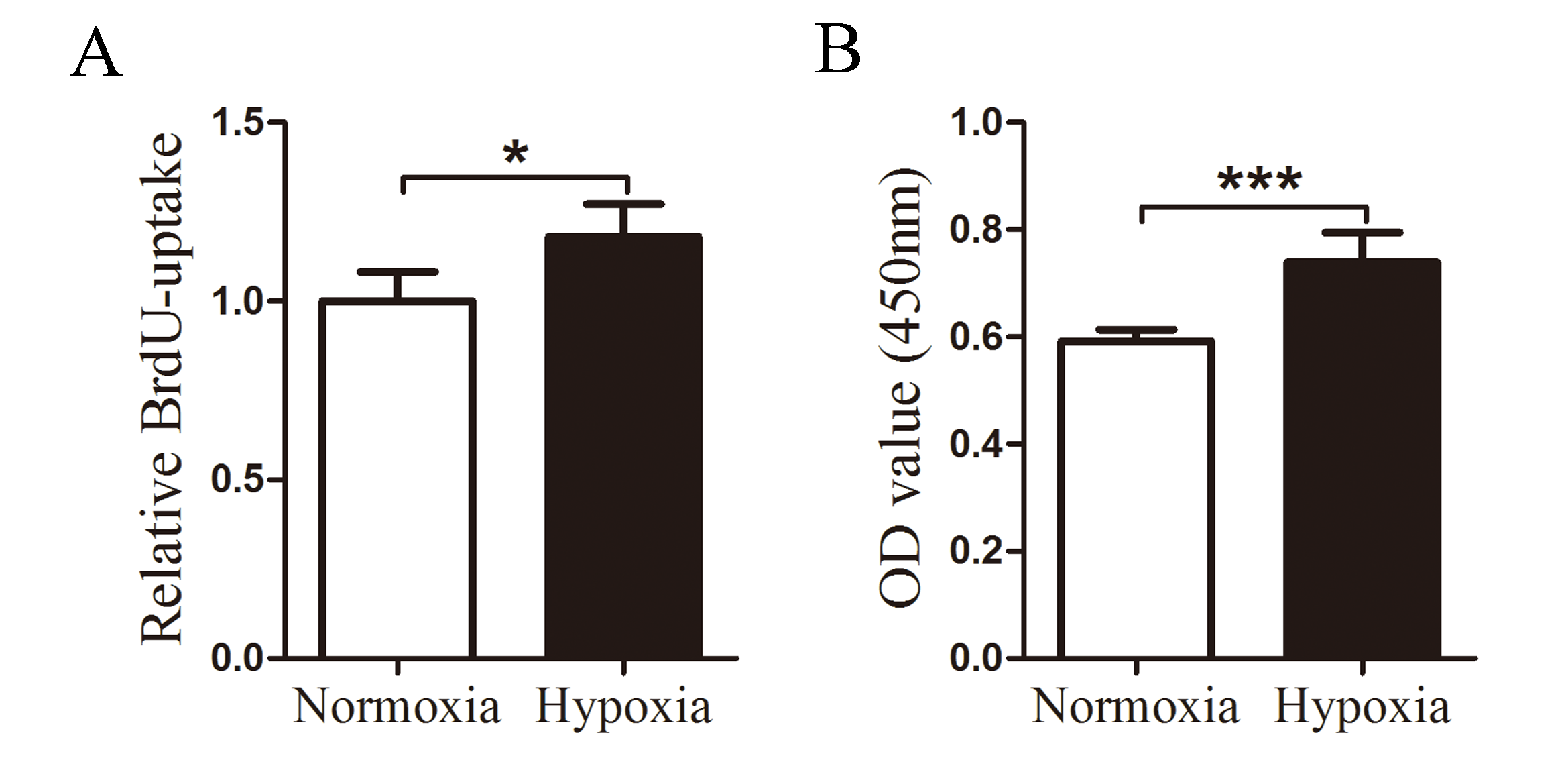

Supplement: Supplementary file 1 — Additional file 1. Characterization of hUCMSCs cultured under hypoxic condition (5% O2). hUCMSCs were subjected to hypoxia for 24 h, A cell proliferation was assessed by using a BrdU-uptake kit (n = 5), B cell viability was evaluated by CCK-8 assay (n = 8). *P < 0.05, ***P < 0.001. [file 13287_2020_2072_MOESM1_ESM.tif]

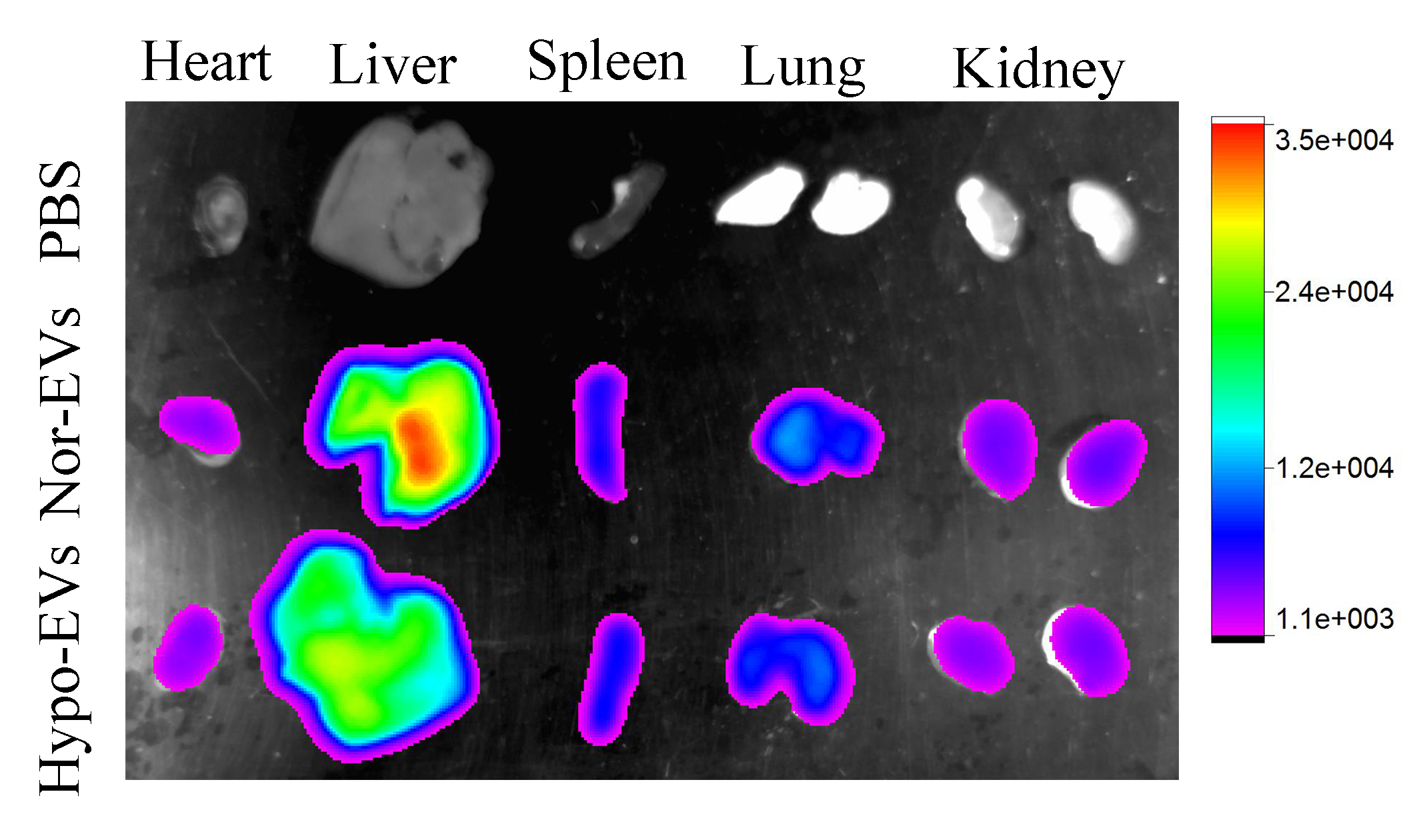

Supplement: Supplementary file 2 — Additional file 2. Biodistribution of DiR-labeled EVs in OVA-mice. Analysis of DiR-labeled EVs (PBS was used as a blank control) after systemic administration was detected using an in vivo imaging system. [file 13287_2020_2072_MOESM2_ESM.tif]

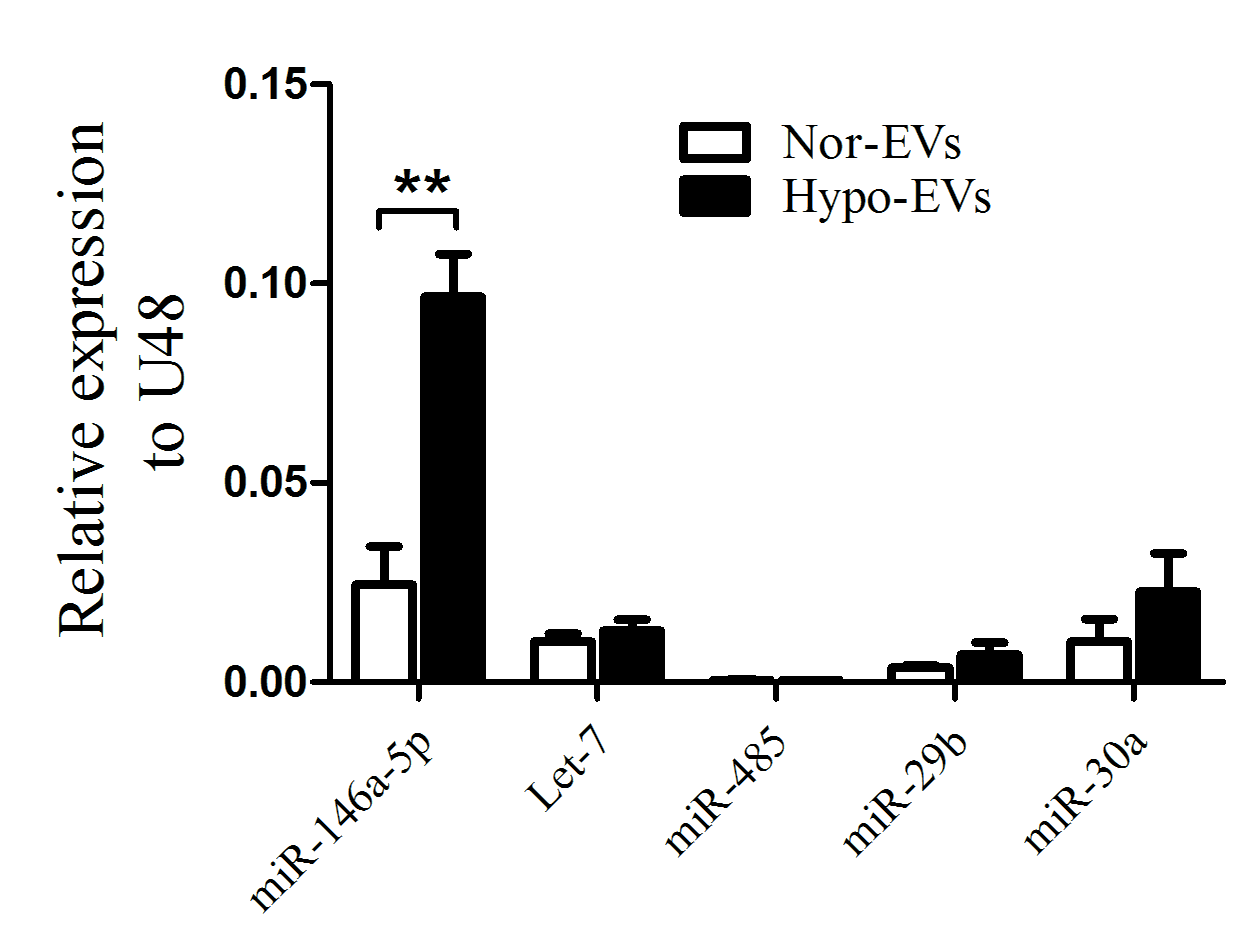

Supplement: Supplementary file 3 — Additional file 3. The expression of miR-146a-5p, let-7, miR-484, miR-29b, and miR-30a in Nor-EVs and Hypo-EVs. The expression levels of the miRNAs were normalized to U48 (n = 3). **P < 0.01. [file 13287_2020_2072_MOESM3_ESM.tif]
